# Frontotemporal dementia: clinical, neuropsychological, and neuroimaging description

**Published:** 2014-09-30

**Authors:** Juan Carlos Rivas Nieto

**Affiliations:** Chief of the Psychiatry Department and Assistant Professor, Universidad del Valle; Psychiatric Hospital Universitario del Valle, Fundación Valle del Lili, Cali, Colombia

**Keywords:** Early onset dementia, Brain imaging, Neuropsychological testing, Rapid functional decline, Differential diagnosis, Praxis preservation

## Abstract

**Objective:**

Describe the relationships between the clinical, neuropsychological, and imaging findings from a group of patients diagnosed with frontotemporal dementia (FTD).

**Methods:**

The clinical histories, cognitive tests, and structural and perfusion brain images of 21 patients of the Psychiatric Hospital Universitario del Valle, Cali, Colombia, were reviewed.

**Results:**

The average age was 59.8 years; the average time for the evolution of disease symptoms was 2.7 years; the most common variant was the behavioral variant; the most common alteration shown through nuclear magnetic resonance (NMR) was frontotemporal atrophy, while the most common alteration shown through single-photon emission computed tomography (SPECT) was frontotemporal hypoperfusion. The most significant result was the normal performance of 61.9% of patients in praxis exams, which was associated with alterations in temporoparietal perfusion in the SPECT images (*p *<0.02). Neither the mini-mental state evaluation nor the Clock Drawing Executive Test (CLOX) served as screening tests.

## Abstract (es)

**Objetivo:**

Describir la relación entre los hallazgos clínicos, neuropsicológicos e imagenológicos en un grupo de pacientes con el diagnóstico de DFT.

**Métodos:**

Se revisaron las historias clínicas, pruebas cognitivas e imágenes cerebrales estructurales y de perfusión de 21 pacientes del Hospital Psiquiátrico Universitario del Valle, Cali, Colombia.

**Resultados:**

El promedio de edad fue de 59.8 años, el tiempo de evolución de la enfermedad fue de 2.7 años, la variante más frecuente fue la comportamental, la alteración más frecuente en la RMN fue la atrofia frontotemporal y en el SPECT fue la hipoperfusión frontotemporal. El hallazgo más importante fue el rendimiento normal del 61.9% de los pacientes en pruebas de praxis, la cual se relacionó con alteración en la perfusión temporo parietal en el SPECT (*p* <0.02). El minimental ni el clox sirvieron como pruebas de tamizaje.

## Introduction

Frontotemporal dementia (FTD) is a neurodegenerative disease that affects the frontal and temporal lobes and presents with alterations to memory, language, and behavior 1. It has a prevalence between 15 and 22 in 100,000 people and an incidence between 2.7 and 4.1 in 100,000 people 2 and is considered to be early-onset dementia. A total of 20-25% of cases occur in individuals older than 65 years 1 ^,^ 2.

Patients with FTD live an estimated 6 to 11 years from the onset of symptoms and 3 to 4 years after diagnosis 2, less than the range seen in Alzheimer's disease. FTD carries serious socioeconomic consequences when it presents at an early age 2. The presence of upper motor neuron symptoms is associated with early mortality 2.

According to the criteria established by Neary *et al.*, FTD is classified into three subtypes: the behavioral variant of FTD (bvFTD), semantic dementia (SD), and primary progressive aphasia (PPA) 3,4. BvFTD is more common in men, and PPA is more common in women 1 ^,^ 2; the reason for this distribution is unknown.

The differential diagnosis for FTD is complex, due partly to other forms of dementia sharing the same symptoms 4. Lamarre *et al.* published new diagnostic criteria for bvFTD, which have been shown to be consistent in recent studies 5. Nevertheless, it is hoped that these criteria will be refined with advances that could allow for a better understanding of the physiopathology of the disease and improvements in neuroimaging that could allow better resolution in the lesions that cause the symptoms of FTD 1.

The behavioral variant is the most common of the FTDs 6. It presents with a gradual deterioration of executive function and personality, while visuospatial ability is affected at advanced stages of the disease 7. The most striking alteration is the change in personality presenting as apathy or disinhibition 1; as the disease progresses, patients likewise lose both a sense of personal hygiene and sphincter control 8, followed by the onset of sociopathic and stereotypic actions, changes in eating patterns, and hyperorality 8 ^-^ 10. Neuropsychological tests show a deficit in executive function and in working memory, accompanied by rule violations and confabulation during the assessment 4 ^-^ 11.

The semantic or temporal variant of FTD is characterized by nominal aphasia and behavioral changes in the presence of an asymmetric degeneration in the temporal lobes 11. Patients with left temporal atrophy show a loss of the semantic meaning of certain words, objects, and concepts while preserving fluency, syntax, and prosody 12 ^-^ 14. Neuropsychological exams demonstrate poor performance in image-word association tasks, while episodic memory, visuospatial abilities, and executive function remain relatively intact 13.

Patients with predominantly right temporal atrophy show a behavioral syndrome similar to the one observed in bvFTD 12, 13. The symptoms include compulsive disorders, changes in appetite, insomnia, weight loss, and sexual dysfunction 12 ^,^ 14 ^,^ 15. 

Primary progressive aphasia shows compromise of both phonological and syntactic aspects of language 4 ^-^ 14. It presents with language apraxia, agrammatism, and mild anomia 14. The comprehension of complex syntactic structures is altered, while comprehension is preserved for simple words. Neurological exams show evidence of supranuclear paralysis, signs of parkinsonism, and apraxia of the extremities. Neuropsychological tests reveal mild deficits in working memory and executive function, while episodic memory and visuospatial function remain relatively well conserved. 

Neuropsychological tests are useful for distinguishing FTDs from other types of dementia such as Alzheimer's disease (AD) 16. The results of these tests vary depending on clinical syndromes; nevertheless, the principal alterations are found in working memory and executive function 13 ^-^ 15.

Neuroimaging provides information on both brain structure and function. These techniques have improved the differential diagnosis of FTD and provide more information about the signs of the different syndromes of FTD 16. Nuclear magnetic resonance (NMR) allows one to distinguish the degree of atrophy in gray matter, while single-photon emission computed tomography (SPECT) and positron emission tomography (PET) visualize perfusion and metabolism in different brain regions, respectively 17; patterns of abnormality depend on the different clinical syndromes.

In patients with bvFTD, imaging techniques have revealed that alterations in frontal structures and the insula are predominant in the early stages of the disease; as the symptoms progress, the atrophy extends to posterior structures such as the temporal and parietal lobes 18. Patients with SD demonstrate an asymmetric atrophy and hypometabolism of the temporal lobes, primarily in the anterior and posterior regions, the perirhinal cortex, the hippocampus, and the amygdala 19 ^,^ 20. PPA presents with a similar pattern, except that the left side is the most affected, as are the posterosuperior region of the temporal lobe and the inferior region of the parietal lobes 20 ^,^ 21. 

Diagnosis of FTD is a difficult task because it shares symptoms with other dementia syndromes. Therefore, a correlation is necessary among clinical findings, performance in neuropsychological tests, and neuroimaging results to help improve FTD diagnosis and differentiate between the variants of FTD.

This study describes the clinical, neuropsychological, and imaging findings from a group of patients diagnosed with FTD, with an emphasis on those findings that would facilitate differentiation between this group of patients and patients afflicted with other types of dementias.

## Materials and Methods

A retrospective study was performed. All patients who were examined at the Psychiatric Hospital Universitario del Valle in Cali, Colombia between the years 2005 and 2013 and diagnosed with FTD based on the criteria from Neary *et al.* 4, were included. Patients were selected if they underwent diagnostic neuroimaging and if they had performed neuropsychological tests.

In all cases, demographic data, handedness, type of FTD (bvFTD and PPA), principal symptom from the clinical profile (behavioral changes, memory changes, or both), and comorbidity with psychotic depressive symptoms according to a mental examination were obtained. The mini-mental state examination (MMSE) 22 and the Clock Drawing Executive Test (CLOX) 23 were used as screening methods for cognitive function. Brain morphology was evaluated using computed tomography (CT), NMR (using a Siemens Magnetom 63 SP at 1.5 Tesla), and cerebral blood flow (SPECT) with hexamethylpropyleneamine oxime (HMPAO) (using a GE Infinia gamma camera and a Celerix processing station).

This study was approved and supervised by the Committee of Bioethics of the Psychiatric Hospital Universitario del Valle. In all cases, informed consent was obtained from a relative or legal guardian.

Variables were analyzed with measures of central tendency and of statistical dispersion. In both cases, a 95% confidence interval was used. Tables were created for variable crossover analysis, and the Chi^2^ test was used to find association among the different variables. Statistical processing was completed using SPSS ® version 15.

## Results

A total of 21 patients (10 women) were evaluated, with an average age of 59.8 years (interquartile range (IQR): 44.0-75.6). The average time for the evolution of symptoms of the disease (EVOL) was 2.7 years (IQR: 1.8-3.6). All patients were right-handed.

The most frequent variant of dementia found was the behavioral variant (16 patients), followed by primary progressive aphasia (4 patients) and semantic dementia (1 patient). The most prevalent initial symptom was changes in memory (42.9%). A total of 47.6% of the patients suffered from depressive symptoms (sadness, frequent crying, feelings of hopelessness, futility, and/or guilt, changes in sleep patterns, changes in appetite). There was evidence of psychotic symptoms (behavioral changes, delirium, or hallucinations) in 23.8% of the patients. MMSE data was obtained from 52.4% of the sample, with results within normal limits (a score between 25 and 30). Ten patients (47.6%) showed alterations via the CLOX (Table 1)**.**

**Table 1.**
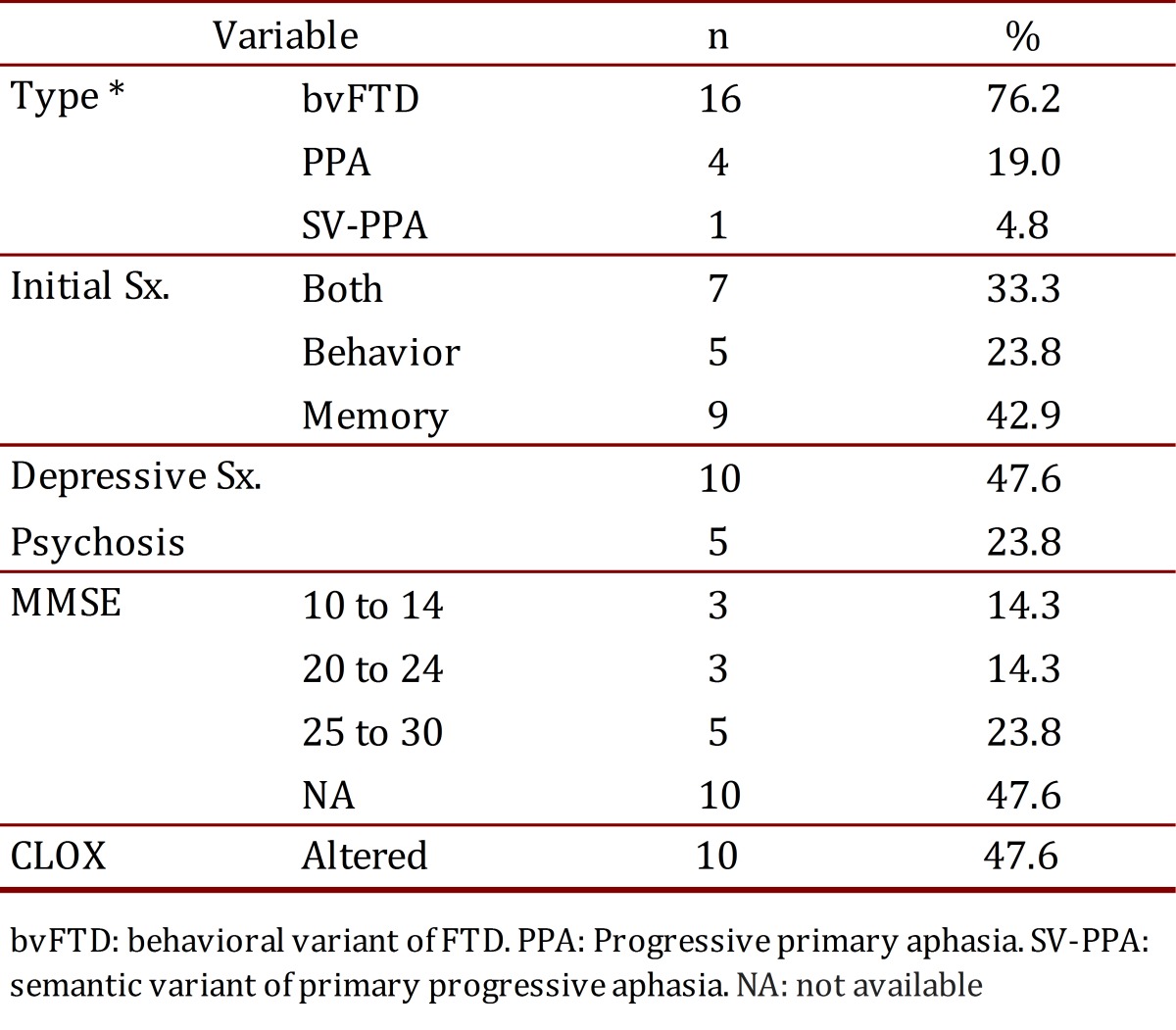
Description of the clinical variables.

The CT was abnormal in 52.4% of the patients; the most frequent abnormality was cortical atrophy (38.1%). The NMR showed frontal and temporal atrophy in 57.1% of the patients (n= 12), followed in frequency by bilateral temporal sclerosis and multiple hyperintensities. The NMR was normal in 33.3% of the patients (n= 7). Using SPECT, we observed that the majority of patients presented with frontotemporal hypoperfusion (42.9%). A total of 71.4% of the evaluated patients presented with a normal EEG (Table 2).

**Table 2.**
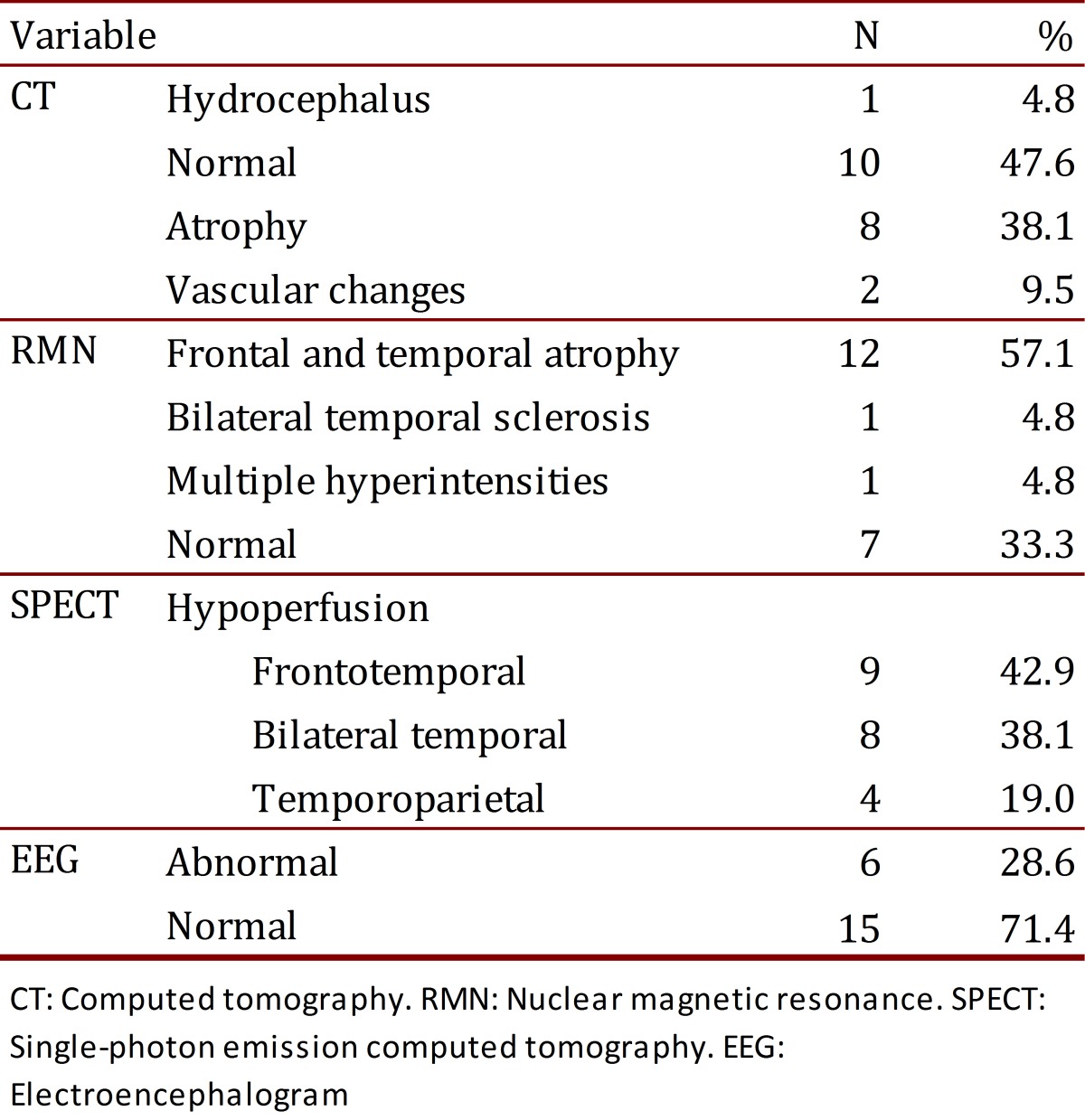
Description of the imaging variables.

In the neuropsychological tests, it was observed that praxis (evaluated via spontaneous drawing with the watch test and reproduction of plane and three-dimensional figures (from Addenbrooke's Cognitive Examination (ACE)) and of the Rey-Osterrieth complex figure) was normal in more than half the patients evaluated (62%). Gnosis, calculation, language, attention, executive functions, and memory were abnormal in the majority of patients tested. A positive relationship was found between changes in praxis and a pattern of temporoparietal hypoperfusion in the SPECT exams SPECT (Table 3).

**Table 3.**
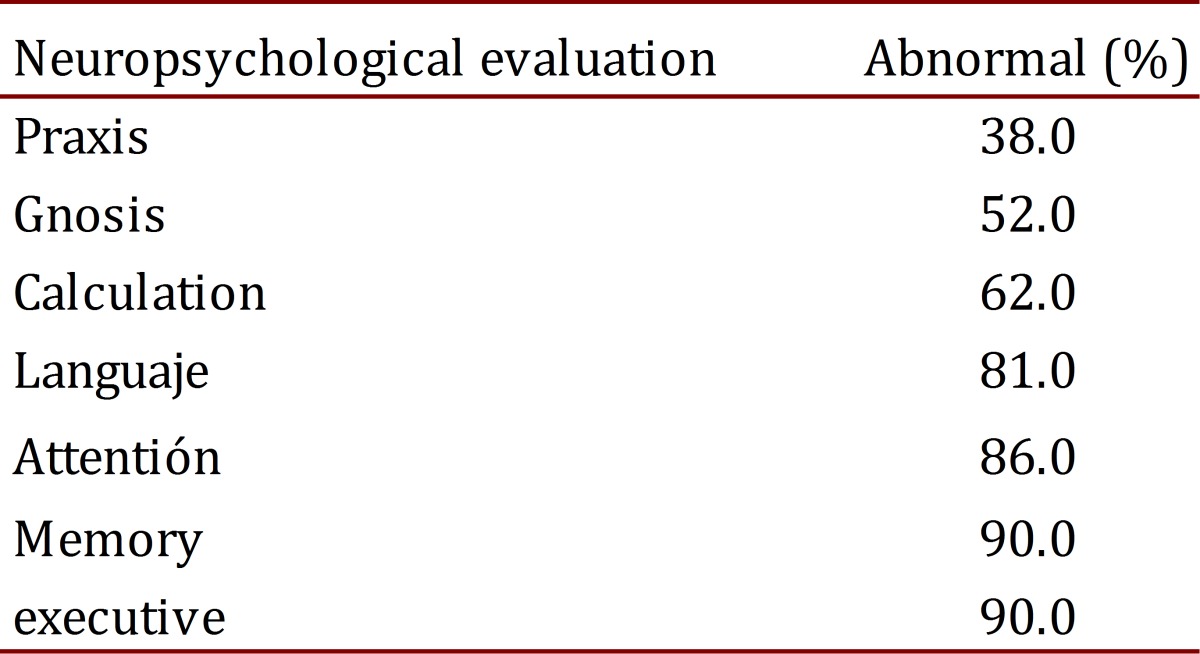
Representation of the neuropsychological variables.

The findings from the SPECT images were related to depressive symptoms (Chi^2^= 7.25, *p*= 0.027). Atrophies in the NMR were related to the type of FTD (Chi^2^= 21.69, *p*= 0.0014). No other association was found among the imaging findings, the clinical results, and the neuropsychological tests (Tables 4 and 5 ).

**Table 4.**
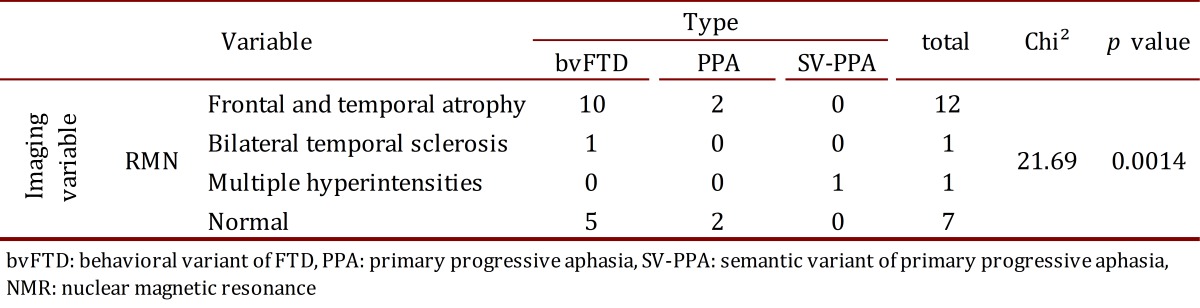
Contingency table between type of FTD and NMR.

**Table 5.**
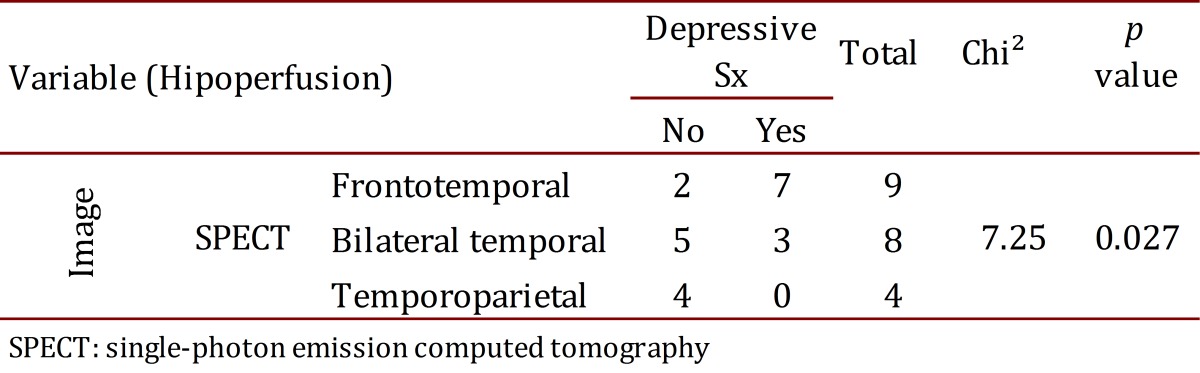
Contingency table between SPECT and depressive symptoms.

A 61.9% prevalence of normal results was found in the neuropsychological tests, with alterations of abnormality found in all elements except for the praxis. 

## Discussion

The diagnosis of FTDs is complex and generally occurs late in the progression of the disease. Once the diagnosis has been made, the patient has already consulted with various medical specialists (internal medicine, neurology, psychiatry) and has been subjected to various unsuccessful exams and treatments. This situation explains the short post-diagnosis survival time 2.

As expected, the sample consisted primarily of individuals under 65 years of age; FTDs constitute one of the significant causes of early-onset dementia and are among the required differential diagnoses in patients presenting with dementia profiles before reaching 65 years of age 24.

The time between the onset of symptoms and diagnosis (2.7 years) accounts for the rapid deterioration described for this kind of dementia. Because memory alterations are a later symptom of the disease, the affective and behavioral symptoms could be tolerated by family members; once the behavioral changes become more striking acts, such as disinhibition and apathy, however, they may seek consultation for the afflicted relative. 

The screening exams were normal in the majority of patients. Both the MMSE and CLOX were within the normal range, consistent with the clinical characteristics of FTDs. These screening methods, useful in late-onset dementias such as Alzheimer's disease, lose validity in FTDs and are replaced by a high degree of clinical suspicion and the use of paraclinical aids.

The presence of depressive symptoms could be evidence of initial symptoms of FTD, as seen in this study's sample. Almost half the patients presented with depressive symptoms, measured with clinical parameters and Yesavage's Geriatric Depression Scale 25. The clinical symptoms of depression are often difficult to distinguish from symptoms associated with FTDs, as dementia symptoms such as apathy and social isolation could be confused with the sadness and anhedonia present in depressive profiles. This confusion results in patients often being subject to various cycles of antidepressants, with the belief that patients are suffering from cases of depression resistant to treatment, consequently delaying a dementia diagnosis. Factors such as clinical suspicion, the lack of response to antidepressants, the absence of cognitive symptoms that normally accompany depression, and alterations in neuropsychological exams and in brain images are all useful elements in a differential diagnosis.

Disinhibition was not a significant symptom in the evaluated patient group. This observation is striking, especially when the majority of patients presented with the behavioral variant of dementia. This absence could be explained through the patients having more symptoms of a depressive nature, where apathy is present, which is also a behavioral symptom. Once again, the presence of symptoms that seem to point to depression arise as a source of confusion, making the diagnostic approximation difficult.

The principal finding of the study was that the patients' performance in the praxis was normal among the majority of the sample, something that has not been reported in previous studies 1 ^,^ 3 ^,^ 8. Additionally, the performance in the praxis was associated with a pattern of temporal and parietal hypoperfusion, a statistically significant result. This observation could be explained by the importance of the parietal lobe in this function and the possibility that the connections from the frontal and temporal lobes to the parietal lobes are relatively conserved in this group of patients, or that their affectation is delayed. A larger sample size is necessary to corroborate this finding.

A retrospective view allows for the possibility that clinical suspicion is a principal factor in the early diagnosis of FTDs. Thus, the presence of symptoms of anxiety, depression and psychosis, and behavior alterations *de novo* in adults should alert the physician to the possibility of an organic factor as an etiological agent. With that suspicion in mind, the low response to antidepressant medications and the presence of symptoms described by Neary and Lamarre require an examination for the possibility of an FTD 3. Finally, diagnosis of FTDs requires the joint use of clinical suspicion, neurological exams, and structural and functional images. Employing all these elements together would lead to greater precision in the diagnosis of dementia with a poor prognosis, not only because of the rapidity of its progression, but especially because of the age when symptoms first present. 

The principal limitations of the study are its retrospective nature, the lack of a control group, and our small sample size. We are currently designing a prospective study; however, due to the low prevalence of this pathology, a multicenter study may be required. 
